# 

**Published:** 1910-04

**Authors:** 


### CONTENTS.

ORIGINAL COMMUNICATIONS—

Some End Results of Non-Removal of the Appendix in 23 Abscess Cases. By R. M. Harbin, M. D., Rome, Ga. ... 1
Stimulation During Anaesthesia. By W. A. Selman, M. D., Atl[illegible]ta, Ga. ... 5
Aneurism.—Report of a Case. By Harry Ainsworth, Thomasville, Ga. ... 11
Empyema.—Report of Cases. By W. D. Gaines, M. D., La Fayette, Ala. ... 15
Pellagra. By D. N. Sage, M. D., Atlanta, Ga. ... 20
Eclampsia. By O. L. Stephenson, M. D., Roanoke, Ala. ... 28
La Grippe. By E. P. Green, M. D., Hickory Flat, Ala. ... 34
The Increasing Frequency of the Use of Narcotic Drugs by Members of the Medical Profeesion and the Probable Reasons for it. By W. C. Ashworth, M. D., Greenesboro, N. C. ... 36

EDITORIALS ... 40

[illegible]STRACTS ... 44

CORRESPONDENCE ... 45

BOOK REVIEWS ... 50

